# Characterization of Leukocytes From HIV-ART Patients Using Combined Cytometric Profiles of 72 Cell Markers

**DOI:** 10.3389/fimmu.2019.01777

**Published:** 2019-08-06

**Authors:** Adrien Leite Pereira, Nicolas Tchitchek, Olivier Lambotte, Roger Le Grand, Antonio Cosma

**Affiliations:** ^1^CEA–Université Paris Sud 11–INSERM U1184, Immunology of Viral Infections and Autoimmune Diseases, IDMIT Infrastructure, Fontenay-aux-Roses, France; ^2^APHP, Service de Médecine Interne-Immunologie Clinique, Hôpitaux Universitaires Paris Sud, Le Kremlin-Bicêtre, France; ^3^Université Paris Sud, Le Kremlin-Bicêtre, France

**Keywords:** high-dimensional cytometry, cytometric profiles merging, HIV infection, chronic inflammation, biomarkers

## Abstract

**Motivation:** Mass cytometry is a technique used to measure the intensity levels of proteins expressed by cells, at a single cell resolution. This technique is essential to characterize the phenotypes and functions of immune cell populations, but is currently limited to the measurement of 40 cell markers that restricts the characterization of complex diseases. However, algorithms and multi-tube cytometry techniques have been designed for combining phenotypic information obtained from different cytometric panels. The characterization of chronic HIV infection represents a good study case for multi-tube mass cytometry as this disease triggers a complex interactions network of more than 70 cell markers.

**Method:** We collected whole blood from non-viremic HIV-infected patients on combined antiretroviral therapies and healthy donors. Leukocytes from each individual were stained using three different mass cytometry panels, which consisted of 35, 32, and 33 cell markers. For each patient and using the CytoBackBone algorithm, we combined phenotypic information from three different antibody panels into a single cytometric profile, reaching a phenotypic resolution of 72 markers. These high-resolution cytometric profiles were analyzed using SPADE and viSNE algorithms to decipher the immune response to HIV.

**Results:** We detected an upregulation of several proteins in HIV-infected patients relative to healthy donors using our profiling of 72 cell markers. Among them, CD11a and CD11b were upregulated in PMNs, monocytes, mDCs, NK cells, and T cells. CD11b was also upregulated on pDCs. Other upregulated proteins included: CD38 on PMNs, monocytes, NK cells, basophils, B cells, and T cells; CD83 on monocytes, mDCs, B cells, and T cells; and TLR2, CD32, and CD64 on PMNs and monocytes. These results were validated using a mass cytometry panel of 25 cells markers.

**Impacts:** We demonstrate here that multi-tube cytometry can be applied to mass cytometry for exploring, at an unprecedented level of details, cell populations impacted by complex diseases. We showed that the monocyte and PMN populations were strongly affected by the HIV infection, as CD11a, CD11b, CD32, CD38, CD64, CD83, CD86, and TLR2 were upregulated in these populations. Overall, these results demonstrate that HIV induced a specific environment that similarly affected multiple immune cells.

## Introduction

Cytometry is an experimental technique used to measure the intensity levels of proteins expressed by cells, at a single cell resolution. However, none of these technologies can currently handle the simultaneous study of more than 50 markers (1). This restriction is particularly limiting to deeply characterize immune cell populations in complex inflammatory diseases, such as HIV infection.

Bioinformatics approaches have been successfully developed and applied to extend the number of observable cell markers per cytometric profiles through the merging of phenotypic information obtained from different cytometry profiles (generated from different cytometry panels). In such approaches, phenotypic information from the different profiles is combined into a single profile based on the expression of a common set of shared markers (2–4). Especially, the CytoBackBone algorithm, which is based on a Nearest Neighbor technique, can be used to extend our vision of complex immune diseases (5). However, no application of these bioinformatics approaches was performed in the context of infectious diseases using mass cytometry data.

HIV infection is still a major sexually transmissible disease. Although detected by different immune receptors, HIV is not cleared by the immune system. A global overview of the cell interaction network triggered in HIV infections is required to understand the immune system dysfunctions and to set up new therapeutic treatments.

HIV preferentially infects CD4^+^ T cells. In the long term, HIV induces a decrease of T cells number both in the blood and in the lymphoid organs. This situation then triggers a severe immune deficiency, that leads to Acquired Immunodeficiency Syndrome (AIDS) (6, 7). The monocyte compartment is also heavily impacted by HIV-infection. Indeed, this disease leads to an increase in the number of CD14^low^ CD16^high^ circulating monocytes (called non-classical monocytes), which is to the detriment of CD14^high^ CD16^low^ monocytes (8). Non-classical monocytes have been characterized as pro-inflammatory cells, as they are able to produce TNF-α, IL-1α, IL1-β, and IP-10 (9–11). In addition, they also overexpress costimulatory molecules such as CD80 and CD86, which again characterizes their activation state (12). Furthermore, the persistence of the virus in the organism results in chronic inflammation, which is also observed in treated HIV-infected patients (13, 14). Interestingly, the persistence of chronic inflammation could lead to an exhaustion of leukocytes (15). This exhaustion is characterized by a delay in the TLR4- or TLR7/8-dependent cytokine productions in monocytes and dendritic cells, as well as by the loss of functional and proliferative capacities of effector T cells. Moreover, several negative regulators of immune activation, such as PD-1, LAG-3, Tim-3, and CTLA-4, are preferentially upregulated on T cells during HIV infection (16–18). Overall, a large set of immune cell populations are involved in this disease.

Many proteins involved in chronic inflammation in HIV-infected subjects have been described (19, 20). Indeed, the literature provides more than 70 cell markers involved in this disease (19, 20). Nonetheless, it is likely that a large number of cell markers are yet to be discovered. Also, a single-cell technology able to simultaneously monitor the expression of a complex network of proteins, involving more than 70 cell markers, is needed to better understand the inflammatory processes generated by HIV infection.

Here, we used the CytoBackBone algorithm (5) to characterize innate and adaptive immune cells obtained from treated HIV-infected patients and healthy donors using combined mass cytometric profiles of 72 cell markers. CytoBackBone is multi-tube cytometry algorithm based on a nearest neighbor approach. Especially, this algorithm uses the notion of acceptable and non-ambiguous neighbors to optimize the quality of the merging of cytometric profiles. Thanks to this notion, the merging performed by CytoBackBone are more stringent and noise-free compared to other approaches. Using this innovative multi-tube cytometry approach, we observed important phenotypic differences between treated HIV-infected patients and healthy donors in the whole leukocyte populations. Especially, we show that monocytes and polymorphonuclear cells (PMNs) are deeply impacted in treated HIV-infected patients. We confirmed these findings using cytometric profiles obtained from a single mass cytometry panel.

## Materials and Methods

### Ethics

This experiment was approved by the Comité de Protection des Personnes (CPP) Ile de France VII, under protocol number PP 14-003. All subjects gave written informed consent to participate in this study.

### Whole Blood Collection

Whole blood from healthy (*n* = 3) and HIV-1 ART-treated non-viremic donors (undetectable plasma RNA, *n* = 3) was collected in lithium heparin tubes by the Etablissement Français du Sang (EFS, Hôpital Saint Louis, Paris, France) and Hôpital du Kremlin Bicêtre, respectively. Information concerning the gender, current age, contamination pathway, viral load, year of detection of the HIV infection, starting year of ARV treatment, and the type and the duration of treatment is provided for each HIV-infected patient in Table 1. The gender and current age of each healthy donor are also provided.

**Table 1:** Characteristics of HIV-infected patients and healthy donors.

**Individuals**	**Gender**	**Current age**	**Contamination**	**Number of CD4^**+**^ T cells (cells/mm^**3**^)**	**CD4^**+**^ T cells nadir (cells/mm^**3**^)**	**Viral load (copie/ml)**	**Detection (age of patient)**	**Treatment started - Updated treatment**	**Current treatment**	**HCV co-infection**
PAT-1	Male	54	Sexual (Homo)	543	204	<40	1994 (32 years)	1997–2015	Tivicay Truvada	No
PAT-2	Female	54	Sexual (Hetero)	1376	167	<40	1991 (26 years)	1994–2014	Isentress Kivexa	No
PAT-3	Female	70	Sexual (Hetero)	404	170	<40	2001 (55 years)	2008–2010	Isentress Truvada	No
PAT-4	Male	51	Sexual (Homo)	1,451	130	<40	1990 (24 years)	1997–2013	Kivexa Norvir Reyataz	No
PAT-5	Male	58	Sexual (Hetero)	324	13	<40	2002 (43 years)	2002–2016	Kivexa Norvir Reyataz	No
PAT-6	Male	48	Sexual (Homo)	744	243	<40	1991 (22 years)	1998–2015	Eviplera	No
HEA-1	Male	48								
HEA-2	Male	63								
HEA-3	Female	28								
HEA-4	Male	59								
HEA-5	Male	56								
HEA-6	Male	56								
HEA-7	Female	59								
HEA-8	Female	25								
HEA-9	Male	27								

*Information about the gender, current age, contamination pathway, viral load, year of detection of the HIV infection, starting year of the ARV treatment, updated year of the ARV treatment, type treatment, the duration of treatments, and the HCV co-infection status were provided for each HIV-infected patient. Information about the gender and the age were provided for each healthy donor. Information presented in this table corresponds to information obtained at the time of the blood samplings*.

To validate the results with a single cytometric panel, blood from the three previous HIV-infected subjects, three new HIV-infected patients (*n* = 6), and six new healthy subjects (*n* = 6) was collected. Information concerning the gender and the current age is provided for each HIV-infected patient and each healthy donor in Table 1. In addition, information concerning contamination pathway, viral load, year of detection of the HIV infection, starting year of ARV treatment, and type and duration of treatment is also provided for each HIV-infected patient.

### Sample Processing for Mass Cytometry Data

Blood samples were processed according to a previously described protocol (21). The cells (from 1 ml blood) were mixed with 10 ml fixation mixture (FM) in 50-ml plastic tubes and incubated for 10 min at 4°C. After centrifugation at 800 x g for 5 min at room temperature (RT), red cells were lysed by adding 10 ml Milli-Q water at RT for 20 min, without agitation. After two washes with 1X DPBS, cells were counted and stored at −80°C in FM at a final concentration of 15 × 10^6^ cells/ml and distributed into aliquots containing 3 × 10^6^ cells. FM used to fix and store the cells was prepared the day before the experiments and conserved at 4°C. The 5% formaldehyde FM solution was prepared from 36% paraformaldehyde (VWR BDH Prolabo, Fontenay-sous-Bois) and contained 18.5% glycerol (Sigma-Aldrich, Lyon, France) in 1X-Dulbecco's phosphate buffered saline (DPBS), without CaCl_2_ or MgCl_2_, pH 7.4 (Gibco by life Technologies, Villebon-Sur-Yvette, France). This solution allowed freezing and recovery of all blood leukocytes, especially polymorphonuclear cells, which are highly labile and cryopreservation-sensitive. Healthy and HIV-infected samples used for the multi-tube 72-marker experiment were cryopreserved for a maximum of 12 days.

### Staining Protocols for Mass Cytometry Data

For each sample, 3 × 10^6^ cryopreserved fixed cells were washed twice with staining buffer [PBS/0.5% BSA, prepared by mixing 1X DPBS modified (Gibco by Life Technologies) with 0.5% BSA (Sigma-Aldrich, Lyon, France)] and labeled with conjugated antibodies according to the following procedure. Cells were incubated at 4°C for 30 min with a mix of the metal-labeled surface antibodies (Abs) in staining buffer. After two washes with 1X DPBS, cells were incubated in fixation solution (PBS/1.6% PFA, prepared by diluting 16% paraformaldehyde (PFA; Electron Microscopy Sciences Hartfield, USA) in DPBS 10X and Milli-Q water) at RT for 20 min and permeabilized with 1X Perm/wash (BD) buffer at RT for 10 min. Staining with metal-labeled intracellular Abs and an iridium nucleic acid intercalator in 1X Perm/Wash was carried out as for extracellular staining, but without a new permeabilization step. Cells were stored overnight with 0.1 μM iridium nucleic acid intercalator in fixation mixture. The following day, cells were washed with Milli-Q water, resuspended in 1 ml Milli-Q water and filtered using a 35-μm nylon mesh cell strainer (BD Biosciences), before the addition of EQ Four-Element Calibration Beads (Fluidigm, San Francisco, USA), according to the manufacturer's instructions. Acquisition of each sample was performed two times in succession on a CyTOF or Helios instrument (Fluidigm). No autosampler was used for sample acquisition. Moreover, the dual sample loop system was used for sample acquisition performed on the CyTOF. CyTOF settings were parametrized following the quality control of the instrument.

### Processing of Mass Cytometry Data

Data were acquired using EQ™ Four-Element Calibration Beads, normalized using Rachel Finck's MATLAB normalizer, and concatenated using the FCS file concatenation tool (Cytobank, Mountain View, CA). Cytometric profiles were manually gated to exclude the EQ™ Four-Element Calibration Beads, select singlets, and gate out non-specific background generated by metal-conjugated Ab binding to eosinophils (22), as represented in Supplementary Figure 1. The arcsinh (cofactor = 5) transformation was applied on marker expressions.

### Merging of Cytometry Profiles Using CytoBackBone

The CytoBackBone algorithm (5) was used to merge cytometric profiles from the different panels. In details, CytoBackBone is an nearest neighbor-based algorithm that combines phenotypic information of different cytometric profiles obtained from different cytometry panels. In our approach, CytoBackBone combines marker expression information of two cells from different cytometric profiles if, and only if, these two cells are *acceptable* and *non-ambiguous* nearest neighbors. In details, each time two cells from different cytometric profiles are identified as acceptable and non-ambiguous nearest neighbors, CytoBackBone merges them and a new cell with a combined phenotype is created. This new cell is added to the resulting cytometric profile and its phenotype corresponds to the average marker expression for the set of common markers. For the non-common markers (i.e., markers that do not belong to the backbone composition), the phenotype of this cell corresponds to the specific marker expression. CytoBackBone is implemented in R and the source code can be found at: https://github.com/tchitchek-lab/CytoBackBone. Each merge realized by CytoBackBone was calculated based on the set of common markers between the cytometric profiles to be merged (CD1c, CD3, CD11c, CD14, CD16, CD19, CD32, CD64, CD66, CD86, CD123, CD141, Granzyme B, and HLADR). A distance threshold of 3 was used for all merging.

### Analysis of Merged Mass Cytometric Profiles

SPADE analyses were generated using the public version of the SPADE R package. viSNE representations were generated based on the Barnes-Hut implementation of the t-SNE algorithm. Dot-plot visualizations were performed using Cytobank software (Mountain View, CA). SPADE analyses were set up and carried out with a down-sampling parameter of 5%. A random pre-downsampling was used to select 67,000 cells from each sample (67,000 corresponded to the number of cells contained in the smallest sample) before the SPADE analysis. Full upsampling was eventually performed after the cell cluster identification by SPADE. Categorical marker heatmaps were generated using the SPADEVizR package (23). Marker expression categories represented in the heatmap were generated based on the intensity expression range of each marker. Marker expression ranges were calculated based on the 5 and 95th percentiles of marker expression. Then, each range was divided into five uniform categories. The categorization of marker expression was computed based on the means of the individual SPADE-expression medians of each marker. These categories represented negative, low, medium, high, and very high marker expression using a color scale from white to dark red, which was used to produce the heatmap. Cell clusters with <50 cells (for the whole dataset) were annotated as unassigned and were colored in gray in the categorical heatmap representation. Indeed, the phenotypes of these clusters cannot be evaluated based on the mean expression of marker expressions. Hierarchical clustering of markers and clusters was performed using the Euclidian distance and was based on the complete linkage method. Statistical analyses of cell cluster abundances were obtained using a non-parametric permutation test (available in the Deducer R package).

## Results

### Phenotypic Characterization of Leukocytes From HIV-Infected Patients and Healthy Donor Using Merged Cytometric Profiles of 72 Cell Markers

To characterize the phenotypes of leukocytes obtained from HIV-infected patients and healthy donors, we used three different mass cytometry panels. Phenotypic information from the different cytometry profiles was combined using the CytoBackBone algorithm. Such multidimensional phenotype characterization was performed based on the simultaneous expression of 72 markers.

We collected whole blood from three non-viremic HIV-infected patients on combined antiretroviral therapies (named PAT-1 to PAT-3) and three healthy donors (named HEA-1 to HEA-3), as indicated in Table 1. Following red cell lysis, cells from each sample were stained using three mass cytometry panels, named #A, #B, #C, which consisted of 35, 32, and 33 markers, respectively (Table 2). These three panels shared 14 common markers.

**Table 2:** Antibodies and cell markers used to create combined mass cytometry profiles including 72 cell markers.

	**Panel #A**		**Panel #B**		**Panel #C**	
**Metals**	**Antibodies**	**Clones**	**Antibodies**	**Clones**	**Antibodies**	**Clones**
Pr141	**CD66**	TET2	**CD66**	TET2	**CD66**	TET2
Nd142	**HLADR**	L243 (G46-6)	**HLADR**	L243 (G46-6)	**HLADR**	L243 (G46-6)
Nd143	**CD3**	UCHT1	**CD3**	UCHT1	**CD3**	UCHT1
Nd144	**CD64**	10.1.1	**CD64**	10.1.1	**CD64**	10.1.1
Nd145	CD8a	37006	CCR7	150503	CD209	DCN47.5
Nd146	IL-6	MQ2-13A5	NF-ATC	7A6	CD62L	DREG-56
Sm147	Granzyme A	CTLA-3	CD335	9 E 2	CD45	D058-1283
Nd148	IL-1β	H1b-98	IL-4	7A3-3	CD137	4B4-1
Sm149	**CD14**	M5E2	**CD14**	M5E2	**CD14**	M5E2
Nd150	**CD123**	7G3	**CD123**	7G3	**CD123**	7G3
Eu151	IL-8	NAPII	CD107a	H4A3	-	-
Sm152	**CD16**	B73.1	**CD16**	B73.1	**CD16**	B73.1
Eu153	CD23	M-L233	CD154	TRAP1	CXCR3 (CD183)	1C6
Sm154	**CD86**	2,331 (FUN-1)	**CD86**	2,331 (FUN-1)	**CD86**	2331 (FUN-1)
Gd155	**CD32**	2 E1	**CD32**	2 E1	**CD32**	2 E1
Gd156	MIP-1β	D21-1351	CD54	LB-2	CD56	AF12-7H3
Gd158	IP-10	6D4	IL-2	N7.48A	ITGβ7	FIB504
Tb159	TNF-α	MAb11	-	-	Bcl-6	K112-91
Gd160	IL-1α	364/3B3-14	CD69	FN50	CD83	HB15E
Dy161	**CD141**	MAB3947	**CD141**	MAB3947	**CD141**	MAB3947
Dy162	IL-12	C8,6	Ki67	B56	CD279	MIH4
Dy163	**CD1c**	AF5916	**CD1c**	AF5916	**CD1c**	AF5916
Dy164	CXCR4	12G5	CD25	BC96	CD127	HIL-7R-M21
Ho165	TLR2	REA109	CD11a	HI111	IL-10R	3F9
Er166	CCR5	3A9	CD11b	ICRF44	CD27	M-T271
Er167	CD28	CD28.2	CD38	AT1	IgG2c	A23-1
Er168	**CD11c**	B-ly6	**CD11c**	B-ly6	**CD11c**	B-ly6
Tm169	IFN-α	LT27:295	-	-	-	-
Er170	CD45RA	T6D11	IgG2b	27-35	CCL5	2D5
Yb171	IFN-γ	25723	IL-10	JES3-9D7	IgG1 K	MOPC-21
Yb172	CD4	L200	MyD88	RB2101	IL-2RA	24212
Yb173	**Granzyme B**	GB11	**Granzyme B**	GB11	**Granzyme B**	GB11
Yb174	**CD19**	HIB19	**CD19**	HIB19	**CD19**	HIB19
Yb175	IL-1RA	AS17	-	-	IgG2a	R35-95
Yb176	MCP-1	5D3-F7	Perforin	dG9-DTAG9	nfKB	K10-892.12.50
Ir191	-	-	-	-	-	-
Ir193	-	-	-	-	-	-

*The metal isotopes, markers, and antibody clones are indicated for each panel. Backbone markers are indicated in bold*.

For each patient, phenotypic information from various mass cytometry panels was merged using the CytoBackBone algorithm. Thus, for each of the six subjects, we generated a merged cytometric profile of 72 cell markers.

For each cytometric profile, the number of cells obtained before and after merging is shown in Table 3. The average of cells present in merged files was 142,265 cells. Furthermore, cytometric profiles related to healthy subjects were composed of 157,083 merged cells (ranging from 103,088 to 229,058 cells), whereas cytometric profiles related to HIV patients were composed of 127,446 merged cells (ranging from 67,280 to 211,223 cells). Among the three cytometric profiles to combine for each individual, more of 97% of cells included in cytometric profiles having the lowest number of cells have were merged. Thus, the processes of merging were thus efficient.

**Table 3:** Number of events and cells obtained, before and after cell merging, for samples used to characterize specific cell populations from healthy subjects and HIV-infected patients.

**Sample ID**	**Stimulation**	**Profile**	**Number of events**	**Number of cells**	**Number of excluded cells**
HEA-1	CTRL	#A	443,527	237,039	-
HEA-1	CTRL	#B	321,545	192,201	-
HEA-1	CTRL	#C	245,550	139,218	-
HEA-1	CTRL	#A+B+C	-	139,104	151,146
HEA-2	CTRL	#A	464,263	249,773	
HEA-2	CTRL	#B	463,584	249,003	-
HEA-2	CTRL	#C	413,188	229,388	-
HEA-2	CTRL	#A+B+C	-	229,058	40,990
HEA-3	CTRL	#A	304,750	128,604	-
HEA-3	CTRL	#B	243,011	112,246	-
HEA-3	CTRL	#C	219,468	103,360	-
HEA-3	CTRL	#A+B+C	-	103,088	34,946
PAT-1	CTRL	#A	395,659	221,756	-
PAT-1	CTRL	#B	275,968	162,754	-
PAT-1	CTRL	#C	177,213	104,389	-
PAT-1	CTRL	#A+B+C	-	103,834	177,397
PAT-2	CTRL	#A	424,726	254,188	-
PAT-2	CTRL	#B	380,247	229,615	-
PAT-2	CTRL	#C	333,418	211,915	-
PAT-2	CTRL	#A+B+C	-	211,223	62,049
PAT-3	CTRL	#A	132,587	77,907	-
PAT-3	CTRL	#B	108,431	68,757	-
PAT-3	CTRL	#C	110,803	68,749	-
PAT-3	CTRL	#A+B+C	-	67,280	13,573

*The number of cells obtained for each profile before and after cells merging was indicated. The profile #A+B+C represents the combined cytometric profiles obtained by the merging of profiles #A, #B, and #C. For each combined profile, the number of cells excluded during the merging was indicated. Samples with names starting with HEA correspond to healthy subjects, and samples with names starting with PAT correspond to HIV-infected patients*.

### HIV-Infection Induces Deep Phenotypic Modifications in Leukocytes

The SPADE algorithm was then used to identify 500 cell populations in the whole dataset of the six merged cytometric profiles. The 72 markers were used as clustering markers to capture the maximal cell diversity (Supplementary Figure 2A). For each cluster, the expression of CD3, CD11c, CD14, CD16, CD19, CD64, CD66, CD123, Granzyme B, and HLADR was represented (Supplementary Figure 2B).

**Figure 1:**
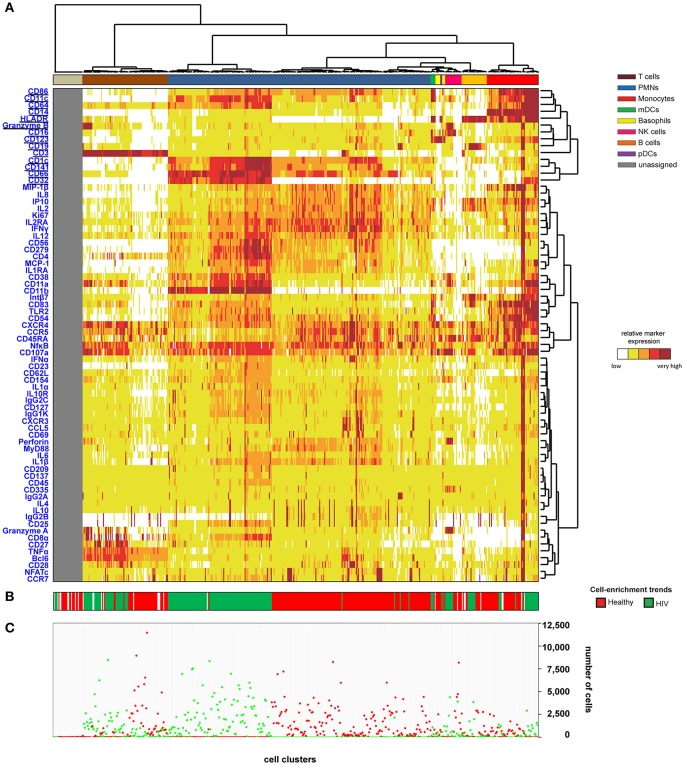
Heatmap representing immune cells from healthy and HIV-infected individuals. Whole blood was collected from three non-viremic HIV-infected patients and three healthy subjects. Following red blood cell lysis, leukocytes of each sample were stained using three mass cytometry panels consisting of 35, 32, and 33 markers. Fourteen markers were shared between the three panels. CytoBackBone was used to generate a merged cytometric profile at the cellular level for each of the six individuals. SPADE analysis was performed to identify 500 cell clusters. The SPADE clustering was generated based on the expression levels of the whole set of 72 cell markers. **(A)** A categorical heatmap representing the relative marker expression for each cluster was generated. Marker expression ranges were calculated based on the 5 and 95th percentile of marker expression. Then, each range was divided into five uniform categories. The categorization of marker expression was computed based on the means of the individual SPADE expression medians of each marker. The categories represent negative, low, medium, high, and bright relative marker expression using a color scale from white to dark red. Hierarchical clustering was performed to visualize clusters with similar expression patterns. Two additional hierarchical clusterings were performed to visualize markers with similar expression patterns: one for common markers and one for non-common markers. Underlined markers correspond to the backbone markers (i.e., markers present in all cytometric panels). Cell clusters were manually annotated on the heatmap. Colors correspond to cell populations. **(B)** The cell-enrichment trend, toward HIV or healthy profiles, is indicated for each cluster. **(C)** The sum of the cells for all individuals and each condition is indicated for each cluster.

We then generated a categorical heatmap representing the relative marker expression for each cluster to interpret the cell-cluster phenotypes (Figure 1). T cell, B cell, PMN, NK cell, monocyte, and dendritic cell clusters were directly annotated on the heatmap according to the expression of CD3, CD11c, CD14, CD16, CD19, CD66, CD123, Granzyme B, and HLADR (Figure 1A). Clusters annotated as unassigned in the heatmap correspond to cell clusters having <50 cells from the whole dataset. In these situations, the number of associated cells is not enough to properly define an MSI for each marker and to associate a population to these cell clusters. The PMN population had the highest diversity and represented more than 50% of the cell clusters. In contrast, the pDC population represented only 0.4% of the cell clusters.

The 500 identified clusters were classified into two categories according to their cell abundance. The first category, named Enrichment Trend Clusters (ETCs), corresponded to clusters for which the cell abundance was specific to either HIV patients or healthy donors (cell abundance fold-change >4). The second category corresponded to other cell clusters (clusters without a specific enrichment trend). The trends of cell-cluster abundance are displayed at the bottom of the heatmap (Figure 1B). The sum of the associated cells from HIV-infected patients or healthy donors is also indicated for each cluster (Figure 1C).

**Figure 2:**
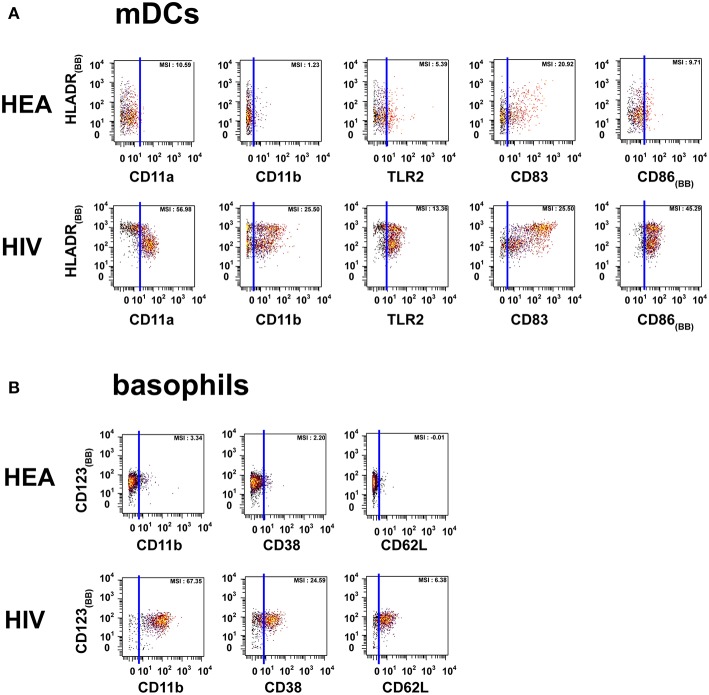
Dot plots representing the phenotypes of mDCs and basophils from HIV-infected patients and healthy donors. Cell clusters corresponding to mDCs were isolated based on the same SPADE analysis presented in Figure 1. The same approach was used to isolate basophils. **(A)** Biplot representations showing the co-expression of HLADR and either CD11a, CD11b, CD83, CD86, or TLR2 by mDC populations. **(B)** Biplot representations showing the co-expression of CD123 and either CD11b, CD38, or CD62L by basophil populations. Blue lines were added to better visualize the difference in marker expression between healthy donors and HIV-infected patients. MSI represents the mean (MSI) of the markers in the X axes. “BB” in subscript indicates backbone markers.

The localization of the ETCs in the heatmap revealed groups of T cell, B cell, PMN, NK cell, and monocyte clusters that were homogeneously enriched either in HIV-infected patients or healthy donors. ETCs specific to the HIV condition were almost exclusively composed of cells from HIV-infected patients, whereas the number of cells from healthy subjects was negligible. Inversely, the enriched clusters for the healthy (HEA) condition were almost exclusively composed of cells from healthy donors, whereas the number of cells from HIV-infected patients was negligible. We identified two pDC clusters: one enriched in the HEA condition, and another in the HIV condition. We compared the phenotypes of CD4 T cells, CD8 T cells, B cells, PMNs, NK cells, monocytes, and pDCs of HIV-infected patients and healthy donors using this heatmap representing the level of 72 cell markers. No specific groups of basophils or mDCs were associated with a specific individual group. The cell clusters of these populations were thus annotated on the SPADE tree and the cells directly analyzed using biplot representations (Figures 2A,B). The CytoBackBone algorithm enabled to observe with a high level of detail differences in the levels of several cell markers. The results and statistical analyses performed here are summarized in Supplementary Table 1.

Most CD8 T cells from healthy subjects were CXCR4^mid^, CD11a^low^, CD11b^neg^, CD27^low^, CD38^neg^, CD83^neg^, Granzyme A^neg^, and Granzyme B^neg/low^. In contrast, CD8 T cells from HIV-infected patients were CXCR4^high^, CD11a^high^, CD11b^mid^, CD27^mid^, CD38^mid^, CD83^mid^, Granzyme A^high^, and Granzyme B^high^ or CXCR4^high^, CD11a^mid^, CD11b^mid^, CD27^high^, CD38^mid^, CD83^mid^, Granzyme A^mid^, and Granzyme B^mid^. Although low in abundance, we observed a CD32^+^ subpopulation in HIV-infected patients. Additionally, CD8 T cells from HIV-infected and healthy individuals were mainly CCR7^neg^, although a set was CCR7^high^ in healthy subjects.

CD4 T cells from healthy subjects were CD11a^low^, CD27^low^, CD38^neg^, CD83^neg^, and Granzyme B^neg/low^. In contrast, CD4 T cells from HIV-infected patients were CD11a^mid^, CD27^mid^, CD38^mid^, CD83^mid^, and Granzyme B^mid^. Although low in abundance, we observed a CD32^+^ subpopulation in HIV-infected patients.

B cells from HIV-infected patients had higher expression of CXCR4, HLADR, CD38, and CD83 than healthy subjects. Indeed, B cells from HIV-infected patients were CXCR4^high^, HLADR^bright^, CD38^mid^, and CD83^high^, whereas B cells from healthy individuals were CXCR4^mid^, HLADR^mid/high^, CD38^neg^, and CD83^neg^.

NK cells from healthy donors were CXCR4^mid^, CD11a^low^, CD11b^neg^, CD38^neg^, Granzyme A^neg^, Granzyme B^neg^, and Perforin^neg^, whereas they were CXCR4^high^, CD11a^high^, CD11b^mid^, CD38^high^, Granzyme A^high^, Granzyme B^high^, and Perforin^high^ in HIV-infected patients.

PMNs from HIV-infected patients were CD11a^high^, CD11b^high^, CD32^high^, CD38^high^, CD64^high^, and TLR2^mid^. However, PMNs from healthy donors were CD11a^low^, CD11b^low^, CD32^neg^, CD38^neg^, CD64^neg^, and TLR2^low^. PMNs from healthy donors also exhibited a set of CCR7^high^ clusters. Basophils from HIV patients were CD11b^high^, CD38^mid^, CD62L^high^, whereas they were CD11b^neg^, CD38^neg^, and CD62L^neg^ in healthy donors.

Monocytes from healthy donors were CD11a^mid^, CD11b^neg^, CD11c^mid^, CD32^neg^, CD38^neg^, TLR2^mid^, and MCP1^neg^, whereas they were CD11a^high^, CD11b^high^, CD11c^high^, CD32^high^, CD38^high^, TLR2^high^, and MCP1^mid/high^ in HIV-infected patients. Although CD64, CD83, CD86, and HLADR were highly expressed by both HIV-infected patients and healthy donors, their levels were higher in HIV-infected patients.

The mDC population can be split into two parts based on the expression of HLADR. However, the modifications of protein expression levels in HIV-infected patients were similar for the two mDC populations. mDCs from HIV-infected patients were CD11a^mid^, CD11b^mid^, CD83^bright^, CD86^bright^, TLR2^high^, and HLADR^bright^, whereas those from healthy donors were CD11a^low^, CD11b^neg^, CD83^mid^, CD86^mid^, TLR2^mid^, and HLADR^high^.

pDCs from HIV-infected patients were CD11b^mid^ and Granzyme B^mid^, unlike pDCs from healthy donors which were CD11b^neg^ and Granzyme B^neg^. Although HLADR was highly expressed in both HIV-infected patients and healthy donors, its level was higher in HIV-infected individuals.

Here, we have described HIV infection-dependent phenotypic changes in T cells, B cells, NK cells, PMNs, monocytes, dendritic cells, and basophils. Overall, the monocyte and PMN populations appeared to be the cell populations most highly affected by HIV infection.

### Confirmation of HIV Infection-Dependent Phenotypic Changes Observed in Monocytes

We next intended to confirm several phenotypic changes obtained with the combined cytometry profiles of 72 markers. To do so, we designed a single mass cytometry panel of 25 cell markers targeting the monocyte population, which was used to stain six new healthy donors (named HEA-4 to HEA-9), three previously used HIV-ART patients (named PAT-1 to PAT-3) and three new HIV-ART patients (named PAT-4 to PAT-6).

The monocyte population was used for validation based on two main reasons. First, monocytes were the most impacted population by HIV and were thus of strong relevance in term of disease characterization. Second, we previously predicted the upregulation of CD11b, CD32, CD64, CD83, CD86, and TLR2 in monocytes from HIV-infected patients. However, these markers were located on different panels. CD32, CD64, and CD86 were present in the backbone, whereas TLR2 was present in panel #A, CD11b in panel #B, and CD83 in panel #C (Table 2). Therefore, the monocyte population represented a good opportunity to validate our results, as these cells expressed these six markers. Thus, we created a single mass cytometry panel of 25 markers that contained these six specific markers (Supplementary Table 2).

Leukocytes from the original three HIV-infected patients (named PAT-1 to PAT-3), three new HIV-infected patients (named PAT-4 to PAT-6), and six new healthy donors (named HEA-4 to HEA-9) (Table 1) were stained using this panel (Supplementary Table 2). After the acquisition of the whole dataset, SPADE analysis was performed. The SPADE analysis was parameterized to obtain 100 clusters using 5% down-sampling, and the clustering based on the levels of CD1c, CD3, CD4, CD8, CD11c, CD14, CD16, CD19, CD32, CD64, CD66, CD86, CD123, CD141, HLADR, and Granzyme B. Then, we annotated the SPADE cell clusters based on the levels of HLADR, CD3, CD11c, CD14, CD16, CD19, CD64, CD66, and CD123. Thereafter, monocytes were computationally isolated. Finally, we visualized the upregulation and co-expression of CD11b, CD32, CD64, CD83, CD86, and TLR2 on monocytes by performing a viSNE analysis (24).

**Figure 3:**
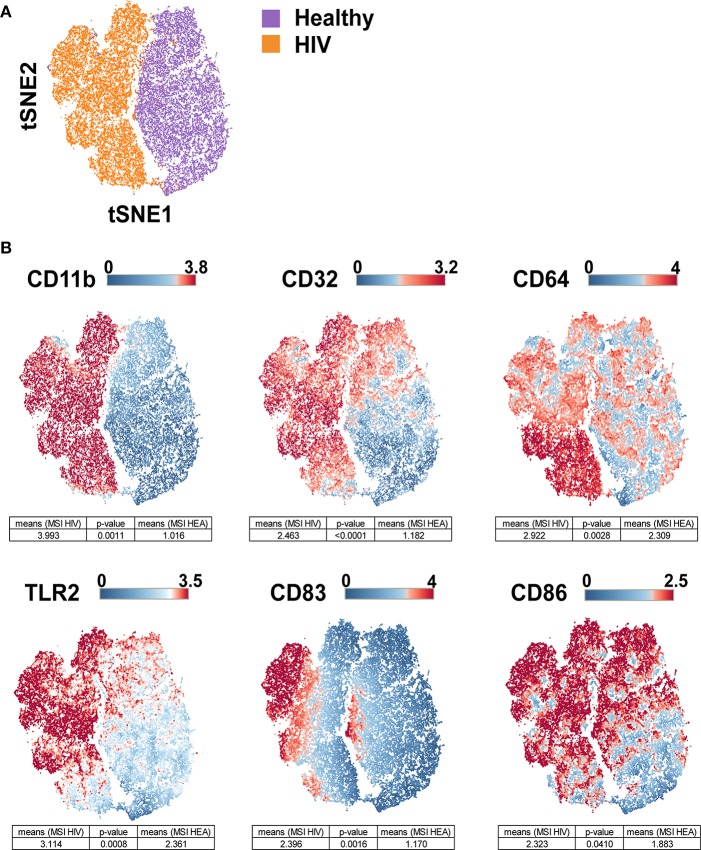
viSNE map representing the phenotype of leukocytes from HIV-infected patients and healthy donors using a single antibody panel. Leukocytes from the three original HIV-infected subjects, three new HIV-infected patients, and six new healthy subjects were collected. SPADE analysis was performed to isolate monocytes. Monocyte cells were computationaly distributed into a two-dimensional space using the viSNE algorithm. Such viSNE representations were generated based on the expressions of CD11b, CD32, CD64, CD83, CD86, and TLR2. **(A)** The viSNE map is colored to represent cells from HIV-infected patients in orange and those from healthy individuals in purple. **(B)** The viSNE map is colored to represent the mean marker expression (MSI) of CD11b, CD32, CD64, CD83, CD86, and TLR2 for each cell. Comparisons of the MSIs from the individual groups were performed using non-parametric permutation tests.

The viSNE visualization, based on the expression of CD11b, CD32, CD64, CD83, CD86, and TLR2, allowed the perfect separation of monocytes from healthy and HIV-infected individuals (Figure 3A). This result demonstrates that the phenotype of monocytes from healthy donors was different from that from HIV-infected individuals. The use of statistical comparisons based on permutation tests allowed us to show that monocytes from HIV-infected patients significantly upregulated (MSI) CD11b (*p* = 0.0011), CD32 (*p* ≤ 0.0001), CD64 (*p* = 0.0028), CD83 (*p* = 0.0016), CD86 (*p* = 0.0410), and TLR2 (*p* = 0.0008) (Figure 3B). In addition, the viSNE visualization allowed us to show that most monocytes from HIV-infected patients were CD11b^high^, CD32^high^, CD64^bright^, CD83^bright^, CD86^bright^, and TLR2^high^. However, there was a subpopulation of monocytes with a CD64^bright^ and TLR2^neg^ phenotype.

**Figure 4:**
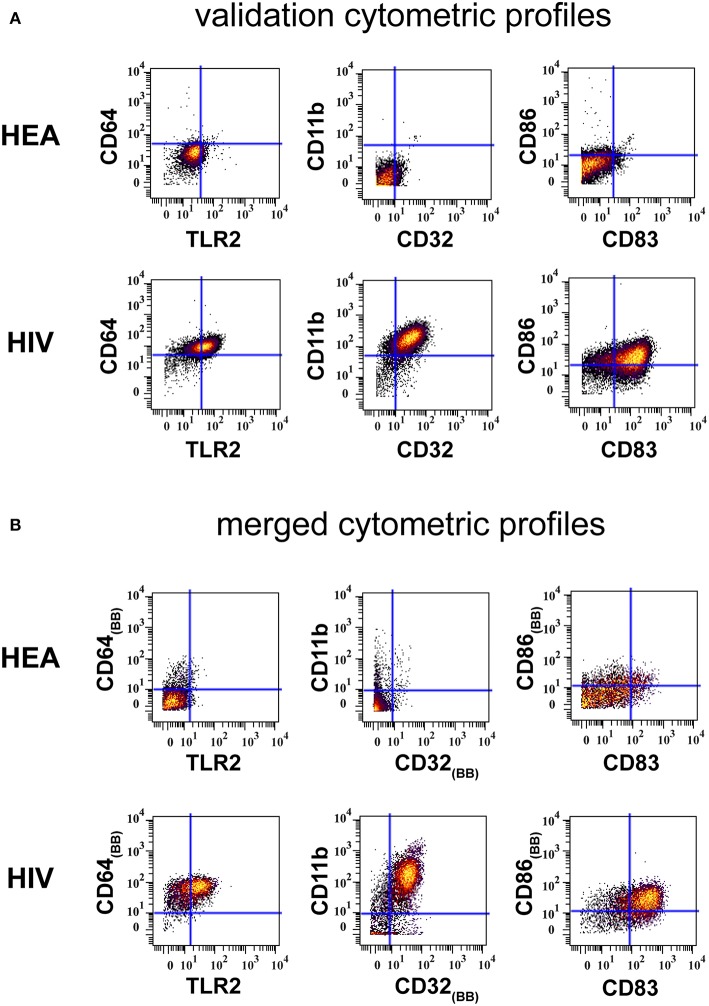
Confirmation of results generated by CytoBackBone. **(A)** Leukocytes were collected from HIV-infected patients and healthy subjects. Cells were stained with a single antibody panel and SPADE analysis was performed to isolate monocytes. The biplot representations show the co-expression of CD64 and TLR2, CD11b and CD32, and CD86 and CD83 in monocytes from patient PAT-1. **(B)** Leukocytes were collected from HIV-infected patients and healthy subjects. Cells were stained with three different antibody panels. Then, CytoBacBone was used to merge the different cytometry profiles. SPADE analysis was then performed to isolate monocytes. The biplot representations show the co-expression of CD64 and TLR2, CD11b and CD32, and CD86 and CD83 in monocytes from patient PAT-1.

Results from the validation experiment were compared to those obtained from the combined mass cytometry profiles using classical dot plots (Figures 4A,B). We observed the same trends of co-expression but no statistical analyses were performed, as these two sets of data were obtained using different panels and were unbalanced in terms of the number of samples.

## Discussion

So far, multi-tube cytometry approaches have not been used in the context of chronic inflammation characterization. In addition to the technical difficulties to perform these experiments using mass cytometry panels, the analysis of such complex data requires further bioinformatics approaches. Here, we addressed these analysis challenges using the SPADE and SPADEVizR pipelines. Even if this study is based on a restricted number of samples from HIV-infected patients, the methodology that we describe here demonstrates that multi-tube cytometry can be applied for characterizing complex diseases.

Here, we characterized the HIV inflammation at an unprocessed phenotypical resolution of 72 cell marker. We detected the upregulation of several proteins in HIV-infected patients. Among them, CD11a and CD11b were upregulated in PMNs, monocytes, mDCs, NK cells, and T cells. CD11b was also upregulated on pDCs. Other upregulated proteins included: CD38 on PMNs, monocytes, NK cells, basophils, B cells, and T cells; CD83 on monocytes, mDCs, B cells, and T cells; and TLR2, CD32, and CD64 on PMNs and monocytes. The monocyte and PMN population appeared to be strongly affected by the HIV infection, as CD11a, CD11b, CD32, CD38, CD64, CD83, CD86, and TLR2 were upregulated in these populations. Overall, these results show that HIV infection induced a specific environment that similarly affected various immune cells in these patients.

Following the detection of Pathogen Associated Molecular Patterns (PAMP) in tissues by cell populations expressing pattern recognition receptors (PRRs), different cytokines and chemokines are produced. The release of these inflammatory proteins leads to the recruitment of innate immune cells, such as neutrophils, monocytes, dendritic cells, and NK cells (25, 26). Whereas, IL-8 and MIP-1β upregulate expression level of adhesion molecules on innate immune cells, cytokines such as IL-1α, IL-6, and TNF-α upregulate the expression levels of selectin molecules on endothelial cells. Interactions between adhesion molecules and selectin molecules trigger the diapedesis mechanism (27–29). In our study, we found that the HIV infection induced an overexpression of adhesion molecules CD11a or CD11b on the surface of neutrophils, monocytes, and dendritic cells. An overexpression of CD11a in NK cells was also detected. Thus, these results suggest that immune cells could be more easily recruited on inflammatory sites.

The HIV infection induces an exhaustion of different innate immune cells, such as neutrophils, monocytes, and dendritic cells. Indeed, we demonstrated that the production of IL-1α, IL-6, and TNF-α were delayed following different TLR stimulations in HIV-infected patients (15). These proteins are essential for the induction of selectin molecules on the surface of endothelial cells. Without these cytokines, leukocytes expressing high levels of CD11a and CD11b cannot adhere to endothelial cells (27–29), which greatly limits the recruitment of immune cells to inflammatory sites.

Innate immunity is strongly activated in primary infection as depicted by the cytokine storm present in the first weeks of HIV infection (30). Numerous types of immune cells are involved in the acute inflammation seen in primary infection. Additionally, these cells are involved in the persistence of the chronic inflammation observed in treated HIV-infected patients. Moreover, a low neutrophil count is associated with the disease progression, a CD4^+^ T cells loss, and a high HIV viral load (31).

Neutrophils contribute to an anti-HIV response through the production of α-defensins, myeloperoxidase, and reactive oxygen species. Neutrophils also participate in the elimination of HIV-infected cells using antibody-dependent cell-mediated cytotoxic processes (ADCC) but are less effective than monocytes and NK cells. Finally, neutrophils and monocytes can perform antibody-dependent cellular phagocytosis of infected cells and immune complexes (32). Thus, phenotype impairment related to PMN and monocytes could be severely harmful. In our study, we observed strong upregulations of CD32 and CD64 on PMN and monocyte populations. CD32 and CD64 are two Fc receptors playing a major role in antibody-mediated processes such as phagocytosis. As phagocytosis is known to be affected by the HIV infection (33, 34), upregulation of these proteins could compensate for this impairment.

CD32 and CD64 also mediate ADCC, which is important in the response against HIV. ADCC is mostly performed by NK cells, but we did not observe an upregulation of Fc receptors on this population. However, monocytes and PMNs also carry out ADCC (35–37). The upregulation of CD32 and CD64 could suggest an increase of ADCC by these cells. Additionally, the increase of expression level of Fc receptors could facilitate the development and maintenance of chronic inflammation. Indeed, the engagement of these receptors leads to the production of cytokines, chemokines, and inflammatory mediators. Consequently, due to the increased expression of these Fc-receptors, associated signaling pathways are probably more expressed. Therefore, this increased expression would result in a greater release of inflammatory molecules, which would promote the maintenance of chronic inflammation.

We also detected an overexpression of MCP-1 on monocytes. MCP-1 is a chemokine that attracts monocytes, lymphocytes, and polymorphonuclear cells. The HIV-NEF protein is known to upregulate the expression of MCP-1 in astrocytes, the most abundant cell type in the brain (38). The detection of MCP-1 on monocytes can be explained by the fact that MCP-1 binds to the CCR2 receptor expressed on monocytes. This could lead to the recruitment of monocytes to the brain through the blood-brain barrier, which could subsequently enhance both the generation of the viral reservoir and neurological complications (39).

Alterations in cytokine expression profiles have reported in treated and non-treated HIV-infected individuals. Here, several cytokines were included in our antibody panels, such as IL-1α, IL-1β, IL-1RA, IL2, IL4, IL6, IL8, IL10, IL12, IP-10, IFN-α, IFN-γ, MIP-1β, TNF-α, and MCP-1. Except for MCP-1, no signal of these cytokines was detected in the different leukocyte populations. This no detection of cytokines is explained by the fact that leukocytes were not stimulated by ligands such as LPS or R848. Without such stimulation, the expression level of cytokines inside leukocytes is too low to be detected.

In our study, no non-classical monocytes (CD14^low^ and CD16^high^) were found in treated HIV-infected patients, neither before nor after the cell merging process. Indeed, all monocytes identified in this study were CD14^high^ and CD16^neg/low^. However, we found that all monocytes up-regulated the expression of CD11a, CD11b, CD11c, CD32, CD38, TLR2, and MCP-1. Thus, these results suggested that both classical and non-classical monocytes were impacted.

Classical myeloid dendritic cells from HIV-infected patients exhibit altered functions including a weak capacity of maturation and inefficient antigen presentation to CD4^+^ T cells (40). Here, we observed a strong up-regulation of CD86 on monocyte and mDC populations, which is a protein essential for antigen presentation. The upregulation of this molecule on mDCs could compensate the dysregulations related to antigen presentation.

Furthermore, we found that the expression of CD83 was higher on monocytes and mDCs from HIV-infected patients compared to healthy subjects. CD83 is a costimulatory molecule and is upregulated after cell activation (41) and maturation (42). CD83-stimulated monocytes suppress T cell immune responses through the production of prostaglandin E2 (43). Upregulation of CD83 also highlights chronic activation of the immune system in ART-treated HIV-infected patients (42).

As pDCs are among the main cells producing IFN-I, they play a main role in the antiviral responses. In HIV-infected patients, the number of pDCs is known to be decreased compared to healthy patients (44). Moreover, the production of cytokines is also impacted by the infection, even in treated HIV-infected subjects (15). In our study, pDCs displayed a hyperactivation phenotype through an up-regulation of CD11b. Thus, we can suppose that pDCs chronically produce IFN-α, which can participate in the increase of myeloid-derived suppressor cells (MDSCs) counts.

Higher transcription of genes encoding TLR2, TLR3, TLR4, TLR6, TLR7, and TLR8 has been described in PBMCs from HIV infected individuals (45, 46). Here, we observed an overexpression of TLR2 in monocytes and PMNs from treated HIV-infected patients. As HIV is able to inhibit signaling pathways induced following the TLRs engagement, the upregulation of these receptors could compensate these inhibitions. In addition, the overexpression of TLRs could have a deleterious effect on the spread of HIV. Indeed, HIV is able to use TLR-dependent signaling pathways to facilitate its replication and propagation (47, 48). Importantly, as the microbial translocation is favored in HIV-infected patients, the multiplication of TLR2 activation could participate in the disease progression. Indeed, commensal bacteria (such as *Lactobacillus acidophilus, Prevotella melaninogenica, Prevotella bivia, and Mycobacterium smegmatis*) enhance the spread of HIV by activating TLR2-dependent signaling (49). Finally, the TLR2 activation is also associated with reactivation of latent viruses (50).

NK cells are critical antiviral effectors of the innate immune system, and deficiencies of this cell population are associated with an increased probability of HIV infection (51, 52). NK cells contribute to the elimination of HIV-infected cells during the acute phase. Indeed, NK cells can release cytotoxic granules, cytokines, and chemokines through the activation of the KIR receptors, the natural cytotoxicity receptors, the C-type lectin receptors, signaling lymphocyte activation family receptors, and Fc receptors.

Although the ADCC is mostly performed by NK cells (51, 52), monocytes and PMNs are also able to do it. Here, we did not observe upregulations of CD32 and CD64 in NK cells from HIV-infected individuals, unlike in monocytes and PMNs. Thus, the capacity of monocytes and PMNs to perform ADCC could be increased in HIV-infected patients.

Here, alterations observed in NK cells from HIV-infected patients characterize a hyperactivation state, which leads to the persistence of chronic inflammation. This inflammation leads to alterations of the NK cells distribution and their functional capacities. Moreover, the chronic inflammation enhances the generation of anergic NK cells, reduces the ADCC activity, and contributes to a poor immunologic reconstitution of CD4 T cells in HIV-infected individuals (53). Finally, we showed that NK cells overexpressed CD38, which is related to progression to AIDS (54).

CD27 is a protein constitutively expressed by naive T cells. This protein plays a main role in proliferation, survival, and differentiation of T cells. Especially, CD27 promotes immune activation and enhances primary, secondary, memory, and recall responses toward viral infections (55). Following the activation of CD4 or CD8 T cells via the TCR/CD3 pathway, the expression of CD27 is increased in T cells. However, after prolonged activation, CD27 becomes gradually switched off (56). Therefore, a high level of CD27 on T cells is often considered as a marker of early activation. Herein, we demonstrated that the expressions of CD27 on CD4 and CD8 T cells were higher in HIV-1 donors compared to healthy donors. These results again describe a persistence of inflammation in HIV-infected patients, which could be associated either to the residual replication HIV or to HIV-dependent inflammatory mechanisms (such as higher microbial translocation). CD27 also plays a key role in the generation and long-term maintenance of T cell responses. The long-term expression of CD27 is associated with impaired maturation of T cells (55). Finally, as the constitutive engagement of CD27/CD70 promotes T cell exhaustion, high levels of CD27 observed in HIV patients could contribute to the loss of T cells effector functions (57).

We found that the expression of CD83 on T cells and B cells from HIV-infected patients was also upregulated compared to healthy donors. The expression of CD83 on CD4^+^/CD25^+^ T cells confers immunosuppressive functions (58), whereas the upregulation of CD83 on murine B cells has a regulatory role in humoral responses (59). Finally, an up-regulation of CD38 was observed on T cells and B cells from HIV-ART patients relative to control individuals, which is associated with HIV disease progression (54). Together, these results support that chronic inflammation could induce important dysfunctions in adaptive immune responses.

NK cells and CD8 T cells naturally express high levels of Granzymes or Perforin. High expressions of Granzyme B and Perforin in CD8 T cells and NK cells are a signature of immune activation, which could be associated here to the persistence of HIV. Indeed, HIV replication is known to induce the production of these cytotoxic proteins in these two cell populations. Importantly, an increase in the number of T cells secreting Granzyme B is associated with reduced viral reservoirs in HIV infection. Thus, HIV infection could limit the secretion of Granzymes and Perforin, and could lead to an accumulation of granules in NK or CD8 T cells. Therefore, the accumulation of granules could lead to the loss of cytolytic activity for NK and CD8 T cells. Finally, the high level of granzymes or perforin observed in HIV-infected patients could be associated with Cytomegalovirus (CMV). Indeed, nearly 90% of HIV-infected patients are CMV positive contrasting with about 60% in healthy donors.

Because HIV-1 infected patients were ART-treated, we suppose that the replication of HIV is not involved in the reported alterations. However, the virus could still play a role, as increasing pieces of evidence point to the persistence of HIV in tissues despite undetectable viral load in plasma (60, 61). Here, we suppose that the phenotypical modifications observed in treated HIV-infected patients are linked to the persistence of inflammation. Indeed, chronic inflammation is associated with a hyperactivation and exhaustion of innate immune cells. This inflammation could be induced by a residual replication of HIV, the presence of HIV in reservoir cells, and an increasing microbial translocation.

Although our combined cytometry panel is composed of 72 cells markers, several important markers for phenotypical annotation of cell subsets are unfortunately absent. Indeed, markers such as CD10, CXCL13, CD278 (follicular T cells), NKG2A, NKp44 (NK cells), T-bet, GATA, RORγt, IL-17 (ILC), or FoxP3 (T regs) were not included in our antibodies panels. Thus, further research must be conducted to extend this approach to characterize all cutting-edge subpopulations involved in HIV.

In the viSNE analysis, monocytes were partly CD64^bright^ and TLR2^neg^ in the single-tube experiment. However, we did not observe this result in the heatmap from the multi-tube 72-marker experiment. The multi-tube 72-marker experiment was performed with three HIV-ART patients (named PAT-1 to PAT-3), whereas the single-tube experiment was performed with six HIV-ART patients (including PAT-1 to PAT-3, but also PAT-4 to PAT-6). After deeper analysis, we found this CD64^bright^ and TLR2^neg^ monocyte population to be detectable in patients PAT-2, PAT-4, PAT-5, and PAT-6. Thus, it was normal to not observe this population on the 72 cell-marker heatmap, as monocytes from patients PAT-1 to PAT-3 were mostly CD64^bright^ and TLR2^high^.

Overall, these analyses lead to the same conclusions: the effect of HIV infection on most innate and adaptive immune cell types are still largely present in the six HIV-infected patients, with a prolonged undetectable viral load and, for most, CD4 T cell counts >500/mm^3^. These results are in agreement with several publications showing persistent chronic immune activation in patients on prolonged ART for whom increased levels of soluble biomarkers (such as sCD14 and IP10) were detected (62, 63).

## Data Availability

Raw and merged cytometric profiles used to characterize the cell phenotypes from healthy subjects and HIV-infected patients as well as the cytometric profiles obtained from the confirmatory single panel are available in the FlowRepository database through the accession IDs: FR-FCM-Z26Z and FR-FCM-Z26Y.

## Ethics Statement

This experiment was approved by the Comité de Protection des Personnes (CPP) Ile de France VII, under protocol number PP 14-003. All subjects gave written informed consent to participate in this study.

## Author Contributions

AL, NT, and AC: conceptualization and methodology, investigation. AL, NT, AC, RL, and OL: validation and writing—review and editing. AL and NT: formal analysis and writing—original draft. AL and OL: resources. AC and RL: funding acquisition. AC, RL, and OL: supervision.

### Conflict of Interest Statement

The authors declare that the research was conducted in the absence of any commercial or financial relationships that could be construed as a potential conflict of interest.
