# Supplementary material for: Characterization of Leukocytes From HIV-ART Patients Using Combined Cytometric Profiles of 72 Cell Markers
Source: Front Immunol. 2019 Aug 6;10:1777. doi: 10.3389/fimmu.2019.01777 (PMC6691046; doi:10.3389/fimmu.2019.01777)
Supplement: Supplementary file 1 [file Data_Sheet_1.PDF]

Characterization of Leukocytes from HIV-ART Patients Using Combined Cytometric Profiles of 72 Cell Markers

Supplementary Materials

Adrien Leite Pereira[1*], Nicolas Tchitchek[1,†,*], Olivier Lambotte[1,2,3], Roger Le Grand[1], and Antonio Cosma[1]

[1]CEA – Université Paris Sud 11 – INSERM U1184, Immunology of Viral Infections and Autoimmune Diseases, IDMIT Infrastructure, 92265 Fontenay-aux-Roses, France

[2]APHP, Hôpitaux Universitaires Paris Sud, Service de Médecine Interne-Immunologie Clinique, 94276 Le Kremlin-Bicêtre, France.

[3]Université Paris Sud, 94276 Le Kremlin-Bicêtre, France.

†Corresponding author

*These authors contributed equally to this work

Corresponding author: Nicolas Tchitchek (nicolas.tchitchek@cea.fr); 18, route du Panorama; 92265 Fontenay-aux-Roses, France.

Supplementary Table

Supplementary Table 1 – Summary of cellular phenotypes from healthy and HIV-infected individuals.

Cell population	Healthy	Markers: Means (MSI healthy); Means (MSI HIV); p-values	HIV
**$CD4^+$ T cells**	CD11a-low , CD27-low , CD38-neg , CD83-neg , and Granzyme B-neg/low	**CD11a: healthy= 0.025; HIV= 0.67; p= 0.0099**<br>**CD27: healthy= -0.03; HIV= 0.81; p= 0.0297**<br>**CD32: healthy= -0.04; HIV= 0.01; p= 0.0198**<br>**CD38: healthy= 0.04; HIV= 0.22; p= 0.0198**<br>**CD83: healthy= 0.01; HIV= 0.36; p= 0.0198**<br>**Granzyme B: healthy= 0.01; HIV= 0.10; p= 0.0198**	CD11a-mid , CD27-mid , CD38-mid , CD83-mid , and Granzyme B-mid<br>+<br>presence of CD32-pos for some cell population subsets
**$CD8^+$ T cells**	CXCR4-mid , CD11a-low , CD11b-neg , CD27-low , CD38-neg , CD83-neg , Granzyme A-neg , and Granzyme B-neg/low	**CXCR4: healthy= 1.41; HIV= 2.36; p= 0.0099**<br>**CD11a: healthy= 0.43; HIV= 1.30; p= 0.0099**<br>**CD11b: healthy= 0.23; HIV= 0.75; p= 0.0396**<br>**CD27: healthy= -0.02; HIV= 1.65; p= 0.0297**<br>**CD32: healthy= -0.02; HIV= 0.13; p= 0.0198**<br>**CD38: healthy= 0.07; HIV= 0.37; p= 0.0198**<br>**CD83: healthy= 0.05; HIV= 0.91; p= 0.0198**<br>Granzyme A: healthy= -0.04; HIV= 0.29; p= 0.0792<br>**Granzyme B: healthy= 0.11; HIV= 0.30; p= 0.0099**	CXCR4-high , CD11a-mid , CD11b-mid , CD27-high , CD38-mid , CD83-mid , Granzyme A-mid , and Granzyme B-mid<br>+<br>presence of CD32-pos for some cell population subsets
**$CD8^+$ T cells**	CXCR4-mid , CD11a-low , CD11b-neg , CD27-low , CD38-neg , CD83-neg , Granzyme A-neg , and Granzyme B-neg/low	**CXCR4: healthy= 1.41; HIV= 2.29; p= 0.0099**<br>**CD11a: healthy= 0.43; HIV= 1.90; p= 0.0099**<br>**CD11b: healthy= 0.23; HIV= 0.75; p= 0.0396**<br>**CD27: healthy= -0.02; HIV=0.75; p= 0.0297**<br>**CD38: healthy= 0.07; HIV= 0.51; p= 0.0198**<br>CD83: healthy= 0.05; HIV= 0.64; p= 0.0594<br>Granzyme A: healthy= -0.04; HIV= 0.67; p= 0.0792<br>**Granzyme B: healthy= 0.11; HIV= 1.27; p= 0.0099**	CXCR4-high , CD11a-high , CD11b-mid , CD27-mid , CD38-mid , CD83-mid , Granzyme A-high , and Granzyme B-high
		**Comparison between two HIV specific T cell populations**<br>CXCR4: p= 0.4752// **CD11a: p= 0.0099**<br>CD11b: p= 0.7525// CD27: p= 0.0198<br>CD38: p= 0.3960// **CD83: p= 0.0396**<br>Granzyme A: p= 0.1584 // **Granzyme B: p= 0.0099**	
**B cells**	CXCR4-mid , HLADR-mid/high , CD38-neg , and CD83-neg	**CXCR4: healthy= 1.32; HIV= 2.36; p= 0.0099**<br>**HLADR: healthy= 3.44; HIV= 4.24; p= 0.0198**<br>**CD38: healthy= 0.05; HIV= 0.56; p= 0.0198**<br>**CD83: healthy= 0.42; HIV= 2.36; p= 0.0198**	CXCR4-high , HLADR-bright , CD38-mid , and CD83-high
**NK cells**	CXCR4-mid , CD11a-low , CD11b-neg , CD38-neg , Granzyme A-neg , Granzyme B-neg , and Perforin-neg	CXCR4: healthy= 0.60; HIV= 1.13; p= 0.2376<br>CD11a: healthy= 0.46; HIV= 0.84; p= 0.2178<br>**CD11b: healthy= 0.18; HIV= 1.16; p= 0.0198**<br>CD38: healthy= 0.04; HIV= 0.47; p= 0.0594<br>Granzyme A: healthy= -0.04; HIV= 0.78; p= 0.2277<br>**Granzyme B: healthy= 0.01; HIV= 0.67; p= 0.0099**<br>**Perforin: healthy=; HIV=; p=**	CXCR4-high , CD11a-high , CD11b-mid , CD38-high , Granzyme A-high , Granzyme B-high , and Perforin-high
**PMN**	CD11a-low , CD11b-low , CD32-neg , CD38-neg , CD64-neg , and TLR2-low	**CD11a: healthy= 0.30; HIV= 1.55; p< 0.0001**<br>**CD11b: healthy= 0.48; HIV= 5.32; p= 0.0099**<br>CD32: healthy= 0.15 ; HIV= 2.35; p= 0.0594<br>**CD38: healthy= 0.34; HIV= 1.03; p= 0.0198**<br>**CD64: healthy= 0.25; HIV= 0.91; p= 0.0198**<br>TLR2: healthy= 0.43; HIV=0.78; p= 0.1386	CD11a-high , CD11b-high , CD32-high , CD38-high , CD64-high , and TLR2-mid
**Basophils**	CD11b-neg , CD38-neg , and CD62L-neg	**CD11b: healthy= 0.27; HIV= 2.29; p= 0.0297**<br>**CD38: healthy= 0.21; HIV=1.60; p=0.0099**<br>**CD62L: healthy= -0.05; HIV= 0.80; p= 0.0198**	CD11b-high , CD38-mid , and CD62L-high
**Monocytes**	CD11a-mid , CD11b-low , CD11c-mid , CD32-neg , CD38-neg , TLR2-mid , MCP1-neg , CD64-high , CD83-high , CD86-high , and HLA-DR- high	**CD11a: healthy= 0.92; HIV= 2.16; p< 0.0001**<br>**CD11b: healthy= 0.35; HIV= 3.69; p= 0.0198**<br>**CD11c: healthy= 1.63; HIV= 2.91; p< 0.0001**<br>**CD32: healthy= 0.34; HIV= 1.93; p= 0.0099**<br>**CD38: healthy= 0.31; HIV= 0.93; p= 0.0198**<br>**TLR2: healthy= 0.96; HIV= 1.84; p= 0.0198**<br>**MCP-1: healthy= 0.33; HIV= 0.89; p< 0.0001**<br>**CD64: healthy= 1.02; HIV= 2.04; p< 0.0001**<br>**CD83: healthy= 1.88; HIV= 3.57; p= 0.0198**<br>**CD86: healthy= 1.25; HIV= 2.10; p= 0.0396**<br>**HLADR: healthy= 2.66; HIV= 3.90; p< 0.0001**	CD11a-high , CD11b-high , CD11c-high , CD32-high , CD38-high , TLR2-high , MCP1-mid/high , CD64-bright , CD83-bright , CD86-bright , and HLA-DR-bright
**mDC**	CD11a-low , CD11b-neg , CD83-mid , CD86-mid , TLR2-mid , and HLADR-high	**CD11a: healthy= 1.19; HIV= 2.86; p= 0.0198**<br>**CD11b: healthy= 0.08; HIV= 1.04; p= 0.0198**<br>**CD83: healthy= 1.87; HIV=3.03; p=0.0198**<br>**CD86: healthy= 1.48; HIV= 2.88; p< 0.0001**<br>HLADR: healthy= 3.79; HIV= 4.52; p= 0.0594<br>**TLR2: healthy= 0.63; HIV= 1.48; p=0.0396**	CD11a-mid , CD11b-mid , CD83-bright , CD86-bright TLR2-high , and HLADR-bright
**pDC**	CD11b-neg , and Granzyme B-neg	**CD11b: healthy= 0.42; HIV= 1.00; p< 0.0001**<br>**Granzyme B: healthy= 0.14; HIV= 0.76; p=0.0198**	CD11b-mid , and Granzyme B-mid

Summary of the cell population phenotypes identified in healthy and HIV-infected individuals. The different marker expression levels were named neg, low, mid, high, or bright. The means of marker expression (MSI) of the two groups were compared using non-parametric permutation tests. Relevant results are shown in bold.

Supplementary Table 2 – Antibodies, metals and cell markers used to validate results obtained from the combined mass cytometric profiles of 72 markers.

Metals	Antibodies	Clones
Pr141	CD66	TET2
Nd142	HLADR	L243 (G46-6)
Nd143	CD3	UCHT1
Nd144	CD64	10.1.1
Nd145	CD8a	37006
Nd146	CD62L	DREG-56
Sm149	CD11a	HI111
Nd150	CD123	7G3
Eu151	CD38	AT1
Sm152	CD16	B73.1
Eu153	CD23	M-L233
Sm154	CD86	2331 (FUN-1)
Gd155	CD32	2 E1
Gd158	Granzyme B	GB11
Gd160	CD83	HB15E
Dy161	CD141	MAB3947
Dy163	CD1c	AF5916
Dy164	CXCR4	12G5
Ho165	TLR2	REA109
Er166	CD11b	ICRF44
Er168	CD11c	B-ly6
Er170	CD14	M5E2
Yb172	CD4	L200
Yb174	CD19	HIB19
Yb175	Perforin	dG9-DTAG9
Ir191	-	-
Ir193	-	-

The metal isotopes and the clone are indicated for each antibody.

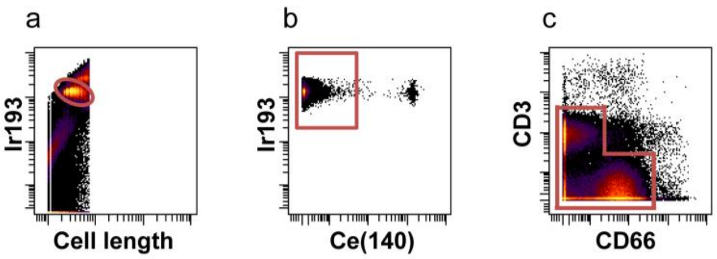

Supplementary Figure 1. Dot plots representing the pre-gating strategy. Samples were manually gated to (**a**) select singlets, (**b**) exclude the EQTM Four-Element Calibration Beads, and (**c**) gate out nonspecific background generated by metal conjugated Ab-binding eosinophils. Singlet cells were gated using Iridium 193 and cell length parameters. EQTM Four-Element Calibration Beads were excluded based on Iridium 193 and Ce(140) parameters. Nonspecific background generated by metal conjugated Ab-binding eosinophils was gated out using CD3 and CD66 parameters.

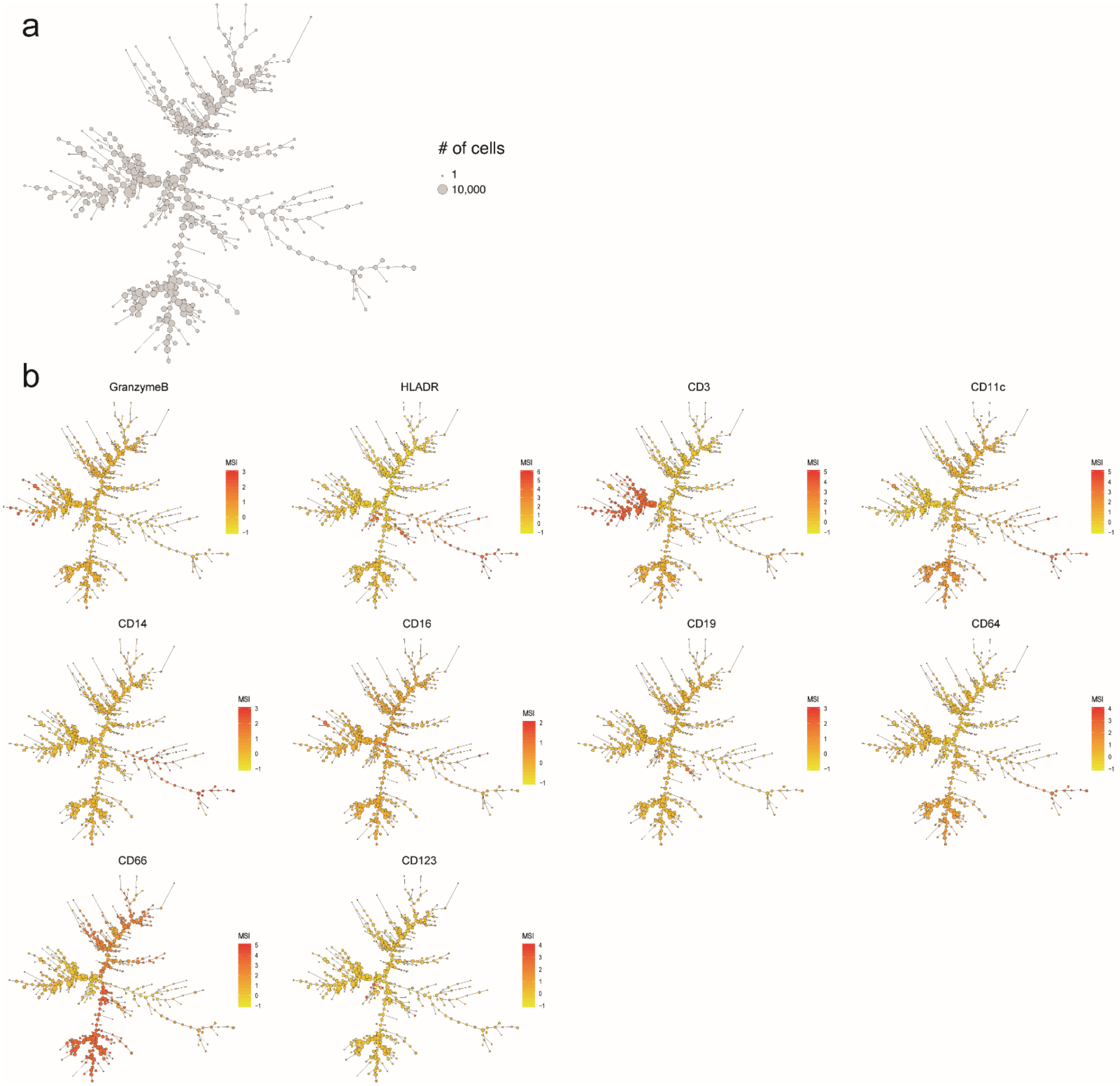

Supplementary Figure 2. SPADE tree representations used to annotate cell clusters. (**a**) SPADE tree representation showing the results of the automatic gating analysis. Each node in the SPADE tree corresponds to a cell cluster and the nodes are linked based on their phenotypical similarities. The size of the nodes is proportional to the number of cells associated with each cluster. (**b**) SPADE tree representations overlaided by the expression levels of CD3, CD11c, CD14, CD16, CD19, CD64, CD66, CD123, Granzyme B, and HLADR to show the consistency of the annotations of T cell, B cell, PMN, NK cell, monocyte, and DC populations.
